# Target discovery screens using pooled shRNA libraries and next-generation sequencing: A model workflow and analytical algorithm

**DOI:** 10.1371/journal.pone.0191570

**Published:** 2018-01-31

**Authors:** Christiane Schaefer, Nikhil Mallela, Jochen Seggewiß, Birgit Lechtape, Heymut Omran, Uta Dirksen, Eberhard Korsching, Jenny Potratz

**Affiliations:** 1 Pediatric Hematology and Oncology, University Hospital Münster, Münster, Germany; 2 Institute of Bioinformatics, Westfälische Wilhelms-Universität Münster, Münster, Germany; 3 Institute of Human Genetics, University Hospital Münster, Münster, Germany; 4 General Pediatrics, University Hospital Münster, Münster, Germany; 5 Department of Hematology and Oncology, Pediatrics III, West German Cancer Center, German Cancer Consortium (DKTK) Center Essen, University Hospital Essen, Essen, Germany; Universitatsmedizin Greifswald, GERMANY

## Abstract

In the search for novel therapeutic targets, RNA interference screening has become a valuable tool. High-throughput technologies are now broadly accessible but their assay development from baseline remains resource-intensive and challenging. Focusing on this assay development process, we here describe a target discovery screen using pooled shRNA libraries and next-generation sequencing (NGS) deconvolution in a cell line model of Ewing sarcoma. In a strategy designed for comparative and synthetic lethal studies, we screened for targets specific to the A673 Ewing sarcoma cell line. Methods, results and pitfalls are described for the entire multi-step screening procedure, from lentiviral shRNA delivery to bioinformatics analysis, illustrating a complete model workflow. We demonstrate that successful studies are feasible from the first assay performance and independent of specialized screening units. Furthermore, we show that a resource-saving screen depth of 100-fold average shRNA representation can suffice to generate reproducible target hits despite heterogeneity in the derived datasets. Because statistical analysis methods are debatable for such datasets, we created ProFED, an analysis package designed to facilitate descriptive data analysis and hit calling using an aim-oriented profile filtering approach. In its versatile design, this open-source online tool provides fast and easy analysis of shRNA and other count-based datasets to complement other analytical algorithms.

## Introduction

RNA interference screens have become a central method in the field of functional genomics to identify critical cancer pathways, molecular drug targets, and their therapeutic synergies [1–8]. In pooled formats and with ready-to-use viral particle shRNA libraries, large-scale screens can now be efficiently performed without expensive liquid- and plate-handling automation, making them accessible to many more laboratories [8–12]. In pooled screens, thousands of different shRNAs are introduced into a cell population, which is then selected for a phenotype of interest. Cells expressing shRNAs that target genes involved in this phenotype are either depleted or enriched compared to a non-selected control population. Independent of the phenotype investigated, this relative change in abundance of individual shRNAs is the basis of pooled screens. In target discovery screens, a relative depletion of shRNAs due to cell death marks these genes as screen hits and potential targets. In order to identify these changes in shRNA abundance, genomic DNA (gDNA) is isolated from selected and control cell populations and integrated shRNA sequences are recovered using PCR. Relative changes are then identified through competitive hybridization of shRNA barcodes to custom microarrays [1–3,13]. More recently and with a higher dynamic range, shRNA abundances have been quantified using next-generation sequencing (NGS), predominantly using Illumina platforms [11–14].

Pooled shRNA screening is a multi-step process, and each step can affect the quality of data and resulting screen hits. Appropriate screen parameters and careful assay development and optimization are therefore critical. For several workflow steps, technical parameters that determine screen performance are now emerging [10–14]. Still, the process of assay development from baseline remains time-intensive and challenging, particularly in rare diseases with a traditional lag in data and methodological guidelines [8]. Apparent challenges lie in the adaptation of novel technologies, such as NGS and large-scale bioinformatics data processing [4,11–15]. In parallel, ‘old’ and basic challenges such as phenotype selection assays, delivery of shRNAs into cultured cells and their on-target gene silencing efficiencies may consume excessive time and resources [8,9,16].

Here we present the assay development of a target discovery screen using pooled shRNA libraries coupled to NGS in a cell line model of Ewing sarcoma, a rare and aggressive cancer of bone and soft tissues. We describe how methods were established and optimized for the entire workflow, from lentiviral transduction to bioinformatics analysis. As part of the analytical algorithm, we developed the ProFED open-source online application to simplify descriptive data analysis including quality control and determination of screen hits in a profile filtering approach.

## Results and discussion

**Fig 1:**
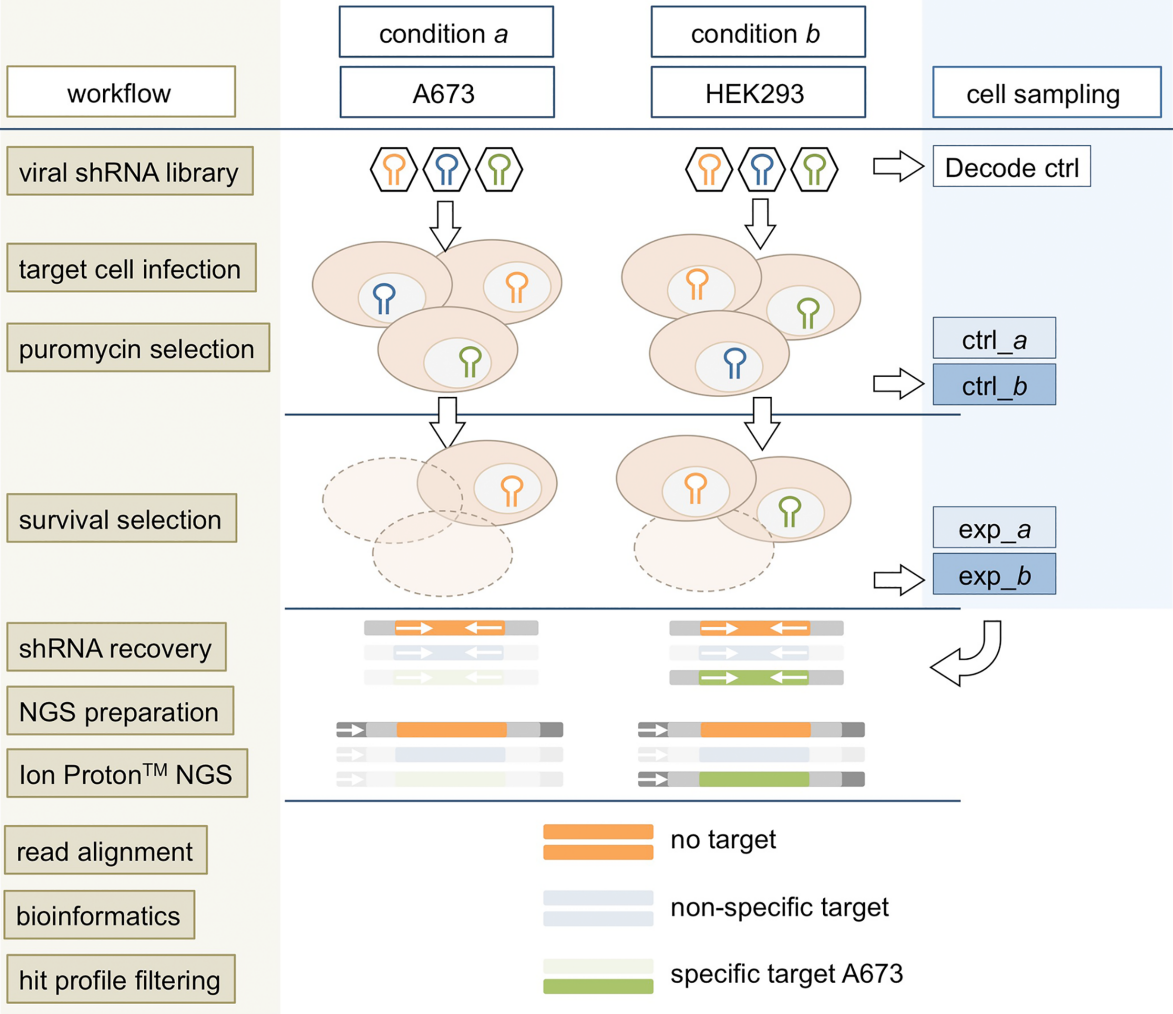
General design and workflow of the pooled shRNA screen. Collected samples of representative cell populations are depicted in blue: ctrl_*a*/*b* = unselected control populations; exp_*a*/*b* = experimental populations selected for phenotype of interest, here cell survival; Decode ctrl = virus particle control.

Our target discovery screen is designed to identify therapeutic targets that are specific to one of two comparative conditions *a* and *b*, such as distinct oncogene expressions, drug treatments, or other synthetic lethal settings; for the purpose of assay development here modeled by distinct cell lines. With an interest in pediatric sarcomas, we chose the A673 Ewing sarcoma cell line (condition *a*) and the HEK293 cell line as a broadly established control (condition *b*). An overview of the general screen design and workflow steps is provided in Fig 1.

### Lentivirus packaging and target cell transduction

The generation of large-scale lentiviral shRNA libraries from arrayed bacterial glycerol stocks requires not only substantial quality control but also liquid handling automation capacities that are unavailable to many research facilities, including ours. We therefore opted for the GIPZ^TM^ lentiviral miR-30 shRNA system based on the G. Hannon and S. Elledge design that is available as commercial ready-to-use lentiviral particles in Decode^TM^ Pooled Lentiviral shRNA Screening Libraries (GE Healthcare Dharmacon) [1,10]. Individual control shRNAs were purchased as glycerol stocks to self-generate these lentiviruses, considering their extensive use during assay development and future target validations. For the delivery of GIPZ shRNA and packaging plasmids into HEK293T packaging cells, both lipid- and calcium phosphate-based transfection methods were evaluated. Since no superior efficiencies of costly lipid-based reagents were observed, subsequent packaging transfections were performed with calcium phosphate as outlined in Materials and Methods.

**Fig 2:**
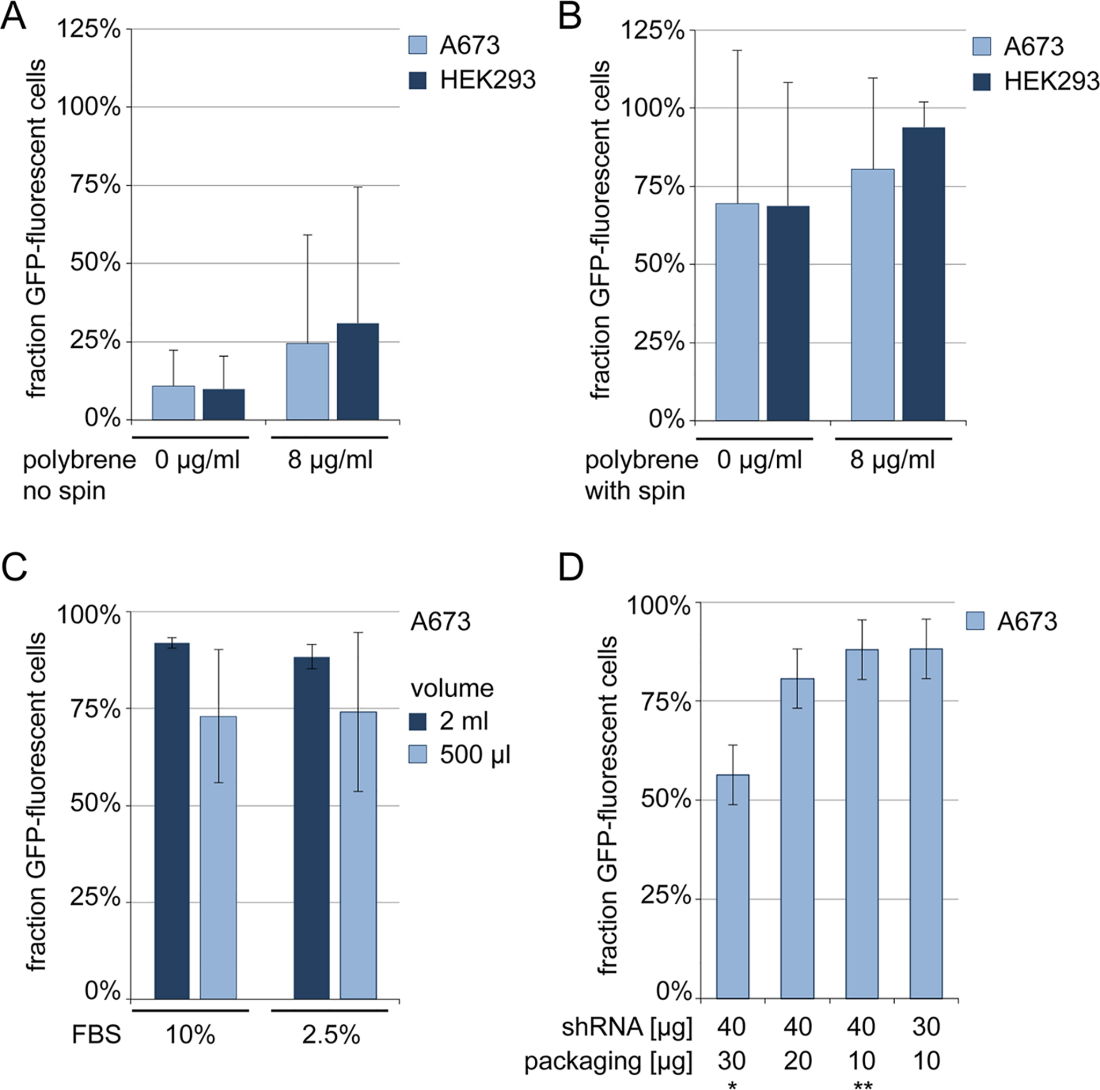
Optimizing transduction conditions for the A673 target cell line. Effects of (A) polybrene and (B) centrifugal spin force on target cell transduction. A673 and HEK293 cells were transduced with 100 μl of non-silencing shRNA lentivirus in a total volume of 2 ml containing 10% FBS. Transduced cell fractions were determined by flow cytometry analysis of GFP expression 72 h later. (C) Influence of transduction volume and serum content was evaluated using 100 μl of LaminA/C lentivirus in spin transductions with 8 μg / ml of polybrene. (D) Effects of viral packaging using distinct ratios of shRNA and packaging plasmids. GAPDH shRNA was packaged in HEK293T cells as indicated and A673 target cells were infected with 100 μl of these viruses using centrifugal spin and 8 μg / ml of polybrene in 2 ml volume containing 10% FBS. Asterisk (*) indicates manufacturer’s recommendation; (**) indicates ratio employed in (A)-(C). All graphs represent mean ±standard deviation (SD) of three independent experiments. Student’s t-test did not reach significance (p < 0.05).

Optimal conditions of target cell transduction are crucial to the success of pooled screens, not only to maximize cost-efficiency of purchased viral particles but also to reproduce technical screen parameters. Transduction conditions were optimized for the A673 Ewing sarcoma cell line and applied to HEK293 cells accordingly. A non-silencing control shRNA and shRNAs targeting LaminA/C and GAPDH were employed and shRNA delivery into target cells was measured by green-fluorescent protein (GFP) expression from GIPZ shRNA plasmids. Representative flow cytometry plots are provided in S1 Fig. In initial transductions, efficiency of virus exposure alone remained low and was insufficiently improved by polybrene (Fig 2A). Double-transductions on consecutive days revealed intolerable cellular toxicity. We found that a crucial factor for improved shRNA delivery into A673 cells was a spin transduction with centrifugal force of virus onto target cells, although experimental variance remained high. Addition of polybrene did not significantly improve viral delivery, but reduced this variance (Fig 2B). Notably, spin transductions can have important logistical consequences for pooled screens, as they may require multiple parallel transductions in smaller plate formats, depending on centrifugation capacities in Biosafety Level 2 containment facilities. Our transductions were therefore performed in 6-well plates. We next evaluated the impact of transduction volume and serum content on transduction efficiency. Although not significant, higher volumes showed a trend towards increased efficiency (Fig 2C) and, together with higher serum content, reduced transduction toxicity upon microscopic evaluation. Our lentivirus transduction protocol thus combined these parameters (Materials and Methods). Of note, proprietary packaging plasmids are a costly aspect of commercial lentiviral packaging systems. Therefore, lentiviruses employed for assay development of target cell transduction were generated using lesser amounts of packaging plasmids than the manufacturer’s recommendation (Material and Methods). Re-evaluation of these viruses using the refined transduction protocol confirmed superior transduction efficiencies (Fig 2D).

### Lentivirus titration and multiplicity of infection

The multiplicity of infection (MOI) describes the ratio of virus-transducing units to target cells and determines the number of shRNA integration events per cell. In pooled screens, this optimal number remains difficult to determine. Whereas low MOIs with delivery of single shRNA copies bear the risk of insufficient shRNA expression and gene silencing, multi-copy shRNA deliveries of high MOIs maximize expression of each shRNA but promote combinatorial phenotypes by simultaneous silencing of multiple genes per cell [16]. While both strategies have been employed successfully with MOIs of 0.3 to 2 [1,2,4,11,17], second-generation shRNA designs now facilitate single shRNA copy gene silencing [10,16] for shRNA-specific phenotypes and we pursued this strategy.

**Fig 3:**
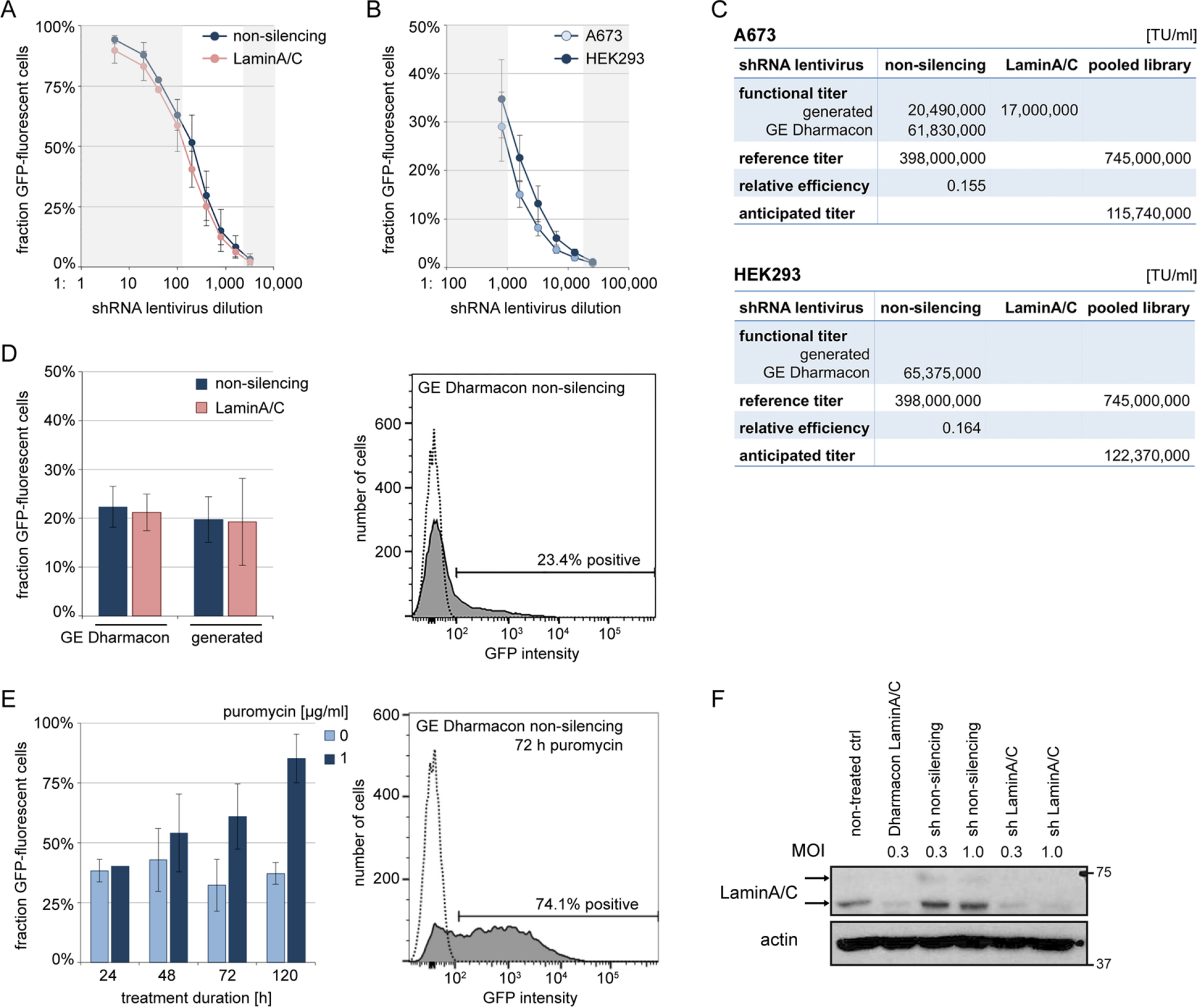
Lentivirus titration and MOI-directed shRNA transduction. (A) Titration curves of non-silencing and LaminA/C shRNA viruses. A673 cells were transduced with serial dilutions of generated lentivirus and GFP expression was analyzed after 72 h by flow cytometry. Functional titers were calculated based on non-shaded dilutions. (B) Titration curves of purchased non-silencing shRNA particles, generated as in (A). (C) Overview of determined functional virus titers [TU / ml] and deduced values. (D) Transduced cell fractions generated with a calculated MOI of 0.3. Left panel: A673 cells were transduced with indicated lentiviruses and GFP expression was analyzed after 72 h. Right panel: Representative flow cytometry plot; shaded graph: transduced cell population; open graph: non-treated control cells. (E) Time-course of puromycin selection. A673 cells transduced with purchased non-silencing shRNA particles in (D) were exposed to puromycin 72 h after transduction. Left panel: GFP-positive cell fractions were determined at time points indicated. Right panel: Representative flow cytometry plot corresponding to that in (D) after 72 h puromycin selection. (F) Western blot of LaminA/C protein expression. A673 cells were transduced as indicated and selected with 1 μg / ml puromycin after 72 h for additional 72 h. Arrows indicate 74 and 65 kDa double-band of LaminA/C. Actin was loading control. All graphs of this figure represent mean ±SD of three independent experiments.

A prerequisite for MOI-directed transductions is that the functional virus titer that is specific to each target cell line and transduction protocol must be determined. To this end, we generated titration curves for non-silencing and LaminA/C shRNA virus in A673 cells (Fig 3A). Of note, a microscopic count of integration events based on GFP-fluorescent single-cell derived colonies was not feasible, because at protocol-defined cell densities fluorescent cells showed a dispersed pattern that did not allow assigning colonies. Quantification was therefore based on flow cytometric analysis of GFP-positive cell fractions. While virus dilutions of 1:5 to 1:100 for both non-silencing and LaminA/C transductions saturated the cell fractions, dilutions as high as 1:200 to 1:1600 were required to achieve an exponential dose-effect relation (Fig 3A). These dilutions were considered for titer calculations (Materials and Methods). Determined functional titers were similar for non-silencing and LaminA/C viruses in A673 (Fig 3C). Subsequently, purchased non-silencing shRNA lentiviral particles were evaluated in both A673 and HEK293 cells (Fig 3B). Their functional titer was only ~3-fold superior to self-generated virus and not significantly different between A673 and HEK293. However, in an optimized protocol for A673, this functional titer remained ~6-fold below the manufacturer’s reference, defining a relative transduction efficiency of ~0.16 for both target cell lines, resulting in an anticipated functional titer of ~12 x10^7^ TU / ml for the pooled shRNA library lentivirus (Fig 3C). To confirm these titers in a reverse approach, we calculated transductions of non-silencing and LaminA/C virus to a MOI of 0.3 (Materials and Methods), which is expected to render 25% of cells with integration events. As shown in Fig 3D for both generated and purchased particles, these transductions reproduced expected GFP-fluorescent cell fractions.

As indicated in Fig 3D, transductions at low MOI leave the majority of cells without shRNA integration and selection is therefore required. After 72 h, allowing for expression of the puromycin resistance marker from integrated GIPZ plasmids, cells were exposed to puromycin. Because single shRNA copy integration must be presumed to confer minimal resistance compared to strategies using higher MOI and shRNA expressions, puromycin was applied at a dose of 1 μg / ml which sufficed to eliminate non-transduced A673 and HEK293 cells (S1 Fig), although a prolonged selection of up to 120 h was required to achieve near-pure populations of shRNA containing cells (Fig 3E). Exemplary flow cytometry plots before and after 72 h of puromycin selection show the shift in transduced cell fractions (Fig 3D and 3E). As demonstrated in Fig 3F, a MOI of 0.3 in cell populations selected with puromycin achieved LaminA/C protein knockdown within 72 h.

Importantly, transduction efficiencies continued to show experimental variance, even when using the same batch of purchased virus particles, resulting in a variance of effective MOI that included values of < 0.3, as indicated by < 25% of GFP-positive cells (Fig 3D). We therefore raised our target MOI for the pooled shRNA library screen to 0.5, accepting ~9% of exposed cells to integrate more than a single shRNA copy.

### Pooled shRNA library screen and cell sampling

The principle of pooled shRNA screening relies on relative changes of each shRNA’s abundance in experimental samples compared to control samples. In loss-of-function cell viability screens, a relative dropout of individual shRNAs due to cell death marks these genes as potential therapeutic targets. Hence, the average shRNA fold representation (the average number of integrations per each shRNA) is a critical screen parameter. Strezoska *et al*. [12] showed that screens at an average 500-fold shRNA representation generated data of higher biological reproducibility, compared to a 100-fold representation. However, higher shRNA fold representation has important logistical and, in the case of purchased libraries, cost-related consequences. Conversely, the authors also demonstrated that shRNAs with robust changes were identified at both screen depths, and successful negative selection screens at 100-fold or less for average shRNA representation have been published [18,19]. Therefore, aiming to explore the irreducible limits of this technology, we conducted this screen at 100-fold shRNA representation.

A Decode Pooled Lentiviral shRNA Library of 4,675 shRNAs targeting 709 human protein kinases was screened in A673 and HEK293 cell lines modeling comparative conditions *a* (A673) and *b* (HEK293). Pooled transductions and puromycin selection were performed according to the parameters defined above (Material and Methods). Anticipating onset of gene silencing, early time-point control samples per each cell line were collected after 48 h of puromycin exposure despite the established prolonged selection time-line (Fig 3E). However, at this point and throughout the screen, cell numbers of collected and cultured populations were adjusted for residual non-transduced cell fractions to maintain 100-fold shRNA representation at all steps (Material and Methods). Early time-point control samples were termed ctrl_*a*/*b* (Fig 1). Remaining cells were cultured for another 5 passages under maintenance of logarithmic growth conditions. The derived experimental samples selected for continued viable cell survival were termed exp_*a*/*b*. An aliquot of Decode library lentiviral particles served as additional non-cellular input control (Decode ctrl); although Sims *et al*. [11] have demonstrated that cellular samples correlated better with plasmid libraries than viral libraries, which was attributed to the viral cDNA preparation step. However, utilizing purchased viral particle libraries, plasmid library controls were not available to us. The entire screen was performed in three independent biological replicates. The first replicate provided test samples for assay development, which were processed prior to the samples of subsequent screen replicates 1 and 2 in all workflow steps.

### shRNA recovery and Ion Proton NGS library preparation

The majority of studies using NGS for deconvolution of shRNA screens have employed Illumina technology [4,13–15]. Here, we evaluated whether the Ion Proton platform could provide a suitable alternative in this short amplicon sequencing application.

**Fig 4:**
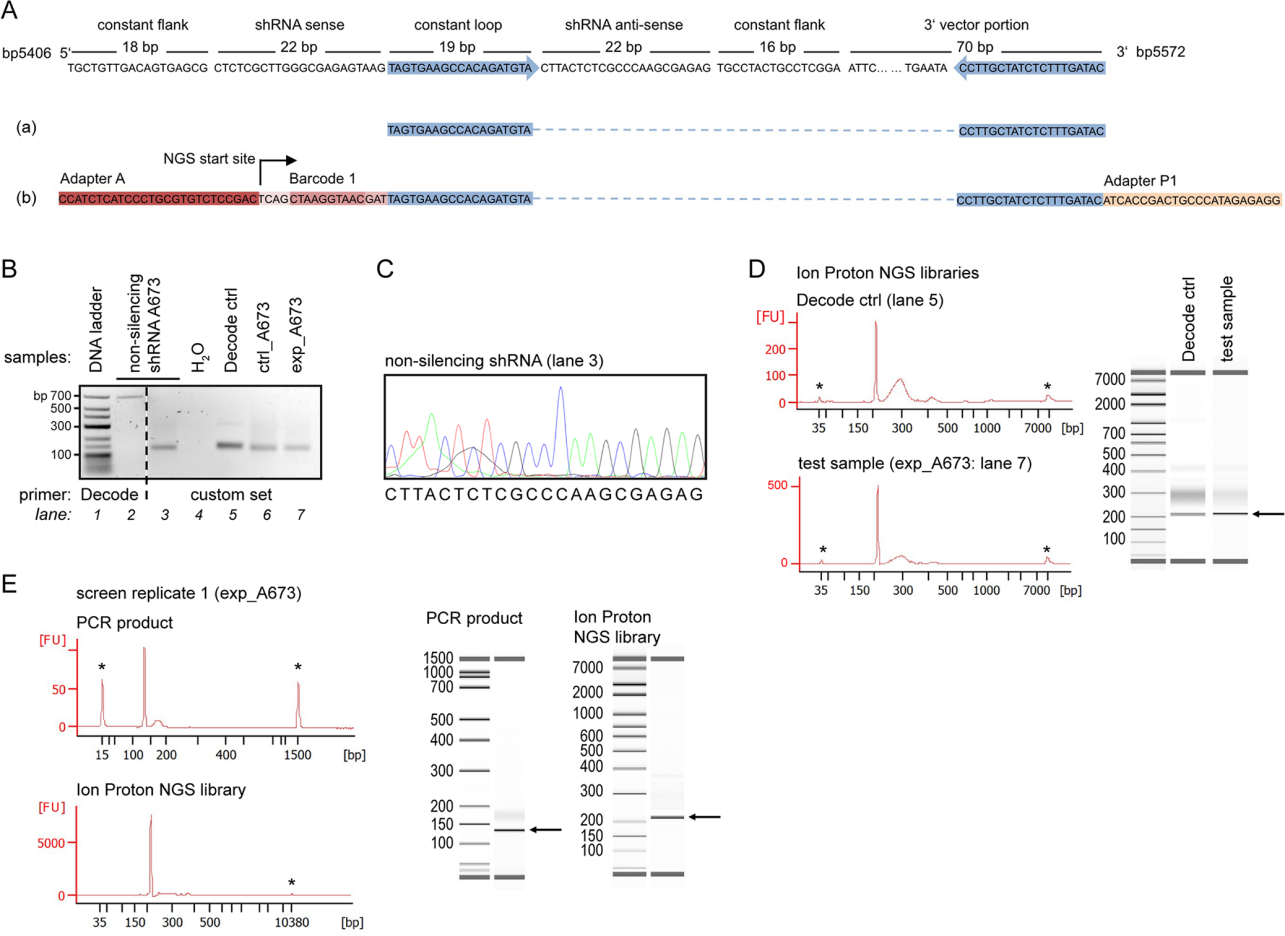
Anti-sense shRNA recovery and Ion Proton NGS library preparation. (A) Excerpt of the GIPZ plasmid sequence with non-silencing shRNA insert indicating primer locations, anti-sense shRNA amplicon (a), and Ion Proton NGS template (b). (B) Gel electrophoresis of anti-sense shRNA PCR products. A673 cells containing non-silencing shRNA (lanes 2–3) were processed identical to A673 test samples (lanes 6–7). For Decode ctrl template (lane 5), cDNA was transcribed from lentiviral particles. Decode Primer PCR (lane 2) followed its manufacturer’s protocol, for custom PCR see Materials and Methods. (C) Sanger-sequence of PCR product recovered from A673 cells that contained non-silencing shRNA (B; lane 3). (D) Bioanalyzer electrophoresis profiles representative of Ion Proton NGS libraries of Decode ctrl and test samples (B; lanes 5 and 7). (E) Bioanalyzer profiles representative of purified PCR products and Ion Proton NGS libraries of screen replicate 1. FU = fluorescent units; asterisk (*) indicates marker; arrow indicates expected fragment size. Profiles of additional samples are provided in S2 Fig.

In the first step, gDNA was isolated from harvested cell populations, and shRNAs integrated into cells’ genomes were recovered by PCR amplification. GE Dharmacon offers proprietary GIPZ-matched Decode PCR Primers that are designed to include NGS barcode tags and adapters but are incompatible with sequencing platforms other than Illumina. We therefore generated an alternative primer set modified from Sims *et al*. [11]. Primers were placed in the constant flanking portions of the GIPZ vector and designed to recover the half-hairpin anti-sense shRNA region, in an amplicon size suitable to Ion Proton NGS (Fig 4A). gDNA of A673 cells transduced with non-silencing shRNA was utilized to optimize PCR reaction composition, template amount and cycling protocols. Fig 4B demonstrates the shRNA amplicon of 127 base pairs (bp) (lane 3) and the 660 bp product of Decode Primers (lane 2) for comparison. Sanger sequencing of lane 3-amplicon confirmed recovery of non-silencing shRNA anti-sense sequence (Fig 4C). Because preservation of the screen’s average shRNA fold representation throughout PCR steps has been shown to improve PCR reproducibility and thereby data quality [11,12], respective amounts of gDNA (Materials and Methods) were amplified in multiple parallel PCR reactions. Products were pooled, purified and confirmed by gel electrophoresis. Fig 4B demonstrates anti-sense shRNA amplicons recovered from A673 test samples (lanes 6–7) and from complementary DNA (cDNA) transcribed from Decode lentiviral particles (lane 5).

Next, Ion Proton-specific NGS templates were prepared for each sample from recovered anti-sense shRNA sequences. To employ NGS cost-effectively, these NGS template libraries can be multiplexed using barcode tags. At a library size of 4,675 shRNAs, a sequencing depth of 1,000 reads per shRNA requires 4.675 million reads per sample. Assuming an Ion Proton platform output of 60–80 million reads of up to 200 bp (according to the manufacturer’s specification), we multiplexed the samples of one screen replicate (ctrl_*a*/*b* and exp_*a*/*b*) plus a Decode ctrl aliquot per run. In this first application of the Ion Proton platform to screen deconvolution, we prepared NGS libraries according to Ion Proton standard protocols with ligation of barcode tags and platform-specific adapters to purified PCR products (Fig 4A). Prior to sequencing, NGS libraries were evaluated by high-resolution Bioanalyzer electrophoresis. In Decode ctrl and test samples, this revealed a two-peaked profile with undefined higher-bp products in addition to Ion Proton NGS libraries (Fig 4D), similar to the profile shown by Sims *et al*. [11] for Illumina NGS libraries. We suspected that PCR-related events such as primer or adapter duplicates, chimeric amplicons, or heteroduplex DNA [13,20] contributed to these higher-bp products. Indeed, analyses of purified PCR products prior to library preparation revealed the same profile (S2 Fig) that was not apparent by standard gel electrophoresis. Also, higher-bp products appeared to be associated with higher total amounts of generated PCR product (S2 Fig), supporting their source in PCR settings near over-amplification. While the exact nature of these products and their impact on NGS efficacy and performance remains undefined, our findings recommend to perform high-resolution Bioanalyzer electrophoresis during optimization and for quality control of PCR settings. However, since in our case the higher-bp side products were much less evident in subsequent screen replicates, both before and after library preparation (Fig 4E and S2 Fig), we did not address this further.

### Sequencing data analysis

**Fig 5:**
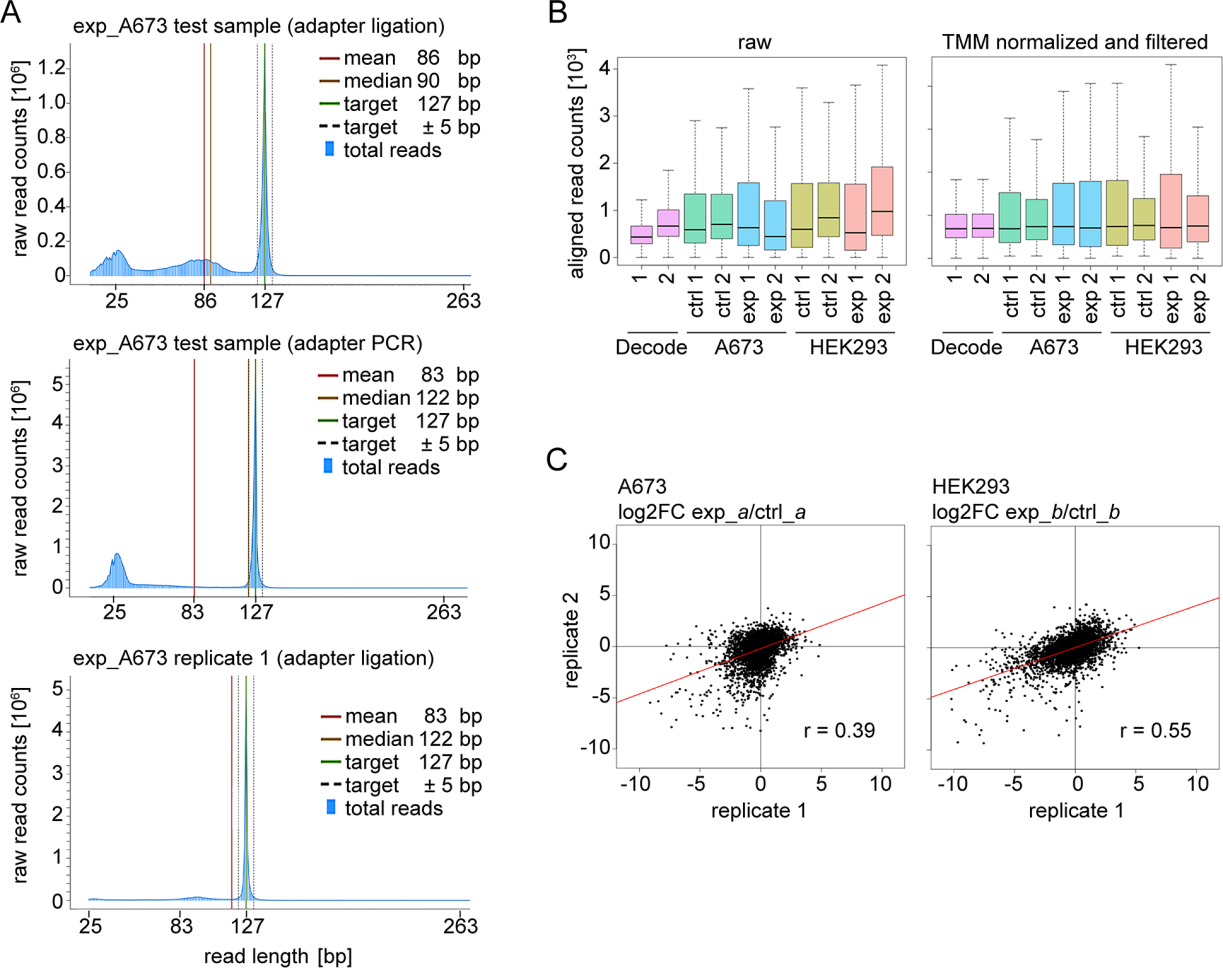
Sequencing data analysis and screen performance. (A) Representative read length histogram of a test sample (exp_A673) in technical replicates of Ion Proton NGS library preparations incorporating adapters via ligation (top panel) or PCR (middle panel), and of its replicate 1 counterpart (bottom panel). (B) Boxplot representation of shRNA read count distribution of raw data (left panel) and of TMM normalized data filtered for shRNAs with ≥ 50 alignments in ctrl_*a*/*b* (right panel). Numbers indicate screen replicates. (C) Biological reproducibility of the relative changes in shRNA abundance. Scatter plots show the correlation of log2 fold changes (FC) of experimental (exp_) relative to control samples (ctrl_) for screen replicates 1 and 2, based on TMM normalized and filtered datasets.

We investigated the Ion Proton sequencer output by read length histograms. Fig 5A (top panel) shows exp_A673 representative of the first sequencing run containing test samples. The peak read length of 127 bp corresponds to the anti-sense shRNA amplicon length. Histogram peaks of longer reads reflecting the higher-bp PCR products of Bioanalyzer profiles were not observed. Instead, histograms showed peaks at shorter read lengths of ±25 and ±85 bp. Upon closer evaluation, these reads represented incomplete sequence read-through with break-off at adapter–constant loop–anti-sense transitions (±25 bp) and in the early constant 3’ vector portion (±85 bp) (compare Fig 4A). In contrast to ±25 bp break-off, ±85 bp reads contained anti-sense shRNA sequence and therefore did not reduce read depth; but their causative events remained elusive. Technical Ion Proton NGS variance likely contributed to incomplete read-through, as both ±25 and ±85 bp break-offs were reduced in a technical sequencing replicate of the same exp_A673 library (S3 Fig). Interestingly, similar to the higher-bp PCR products of Bioanalyzer profiles, read break-offs also were diminished in consecutive screen replicates, where mean read length approached 127 bp (Fig 5A, bottom panel, and S3 Fig). To test whether read break-off was linked to these PCR products or due to NGS library preparation, we evaluated an aliquot of the exp_A673 test sample in an alternative NGS library preparation strategy that incorporated adapters in an additional 16-cycle PCR. Bioanalyzer electrophoresis of this NGS library revealed an increase in higher-bp products consistent with a multiplication of PCR-related events (S3 Fig). However, this did not result in increased ±85 bp break-off but the histogram revealed improved read-through (Fig 5A, middle panel), indicating Ion Proton library preparation as critical step for sequence read-through. Still, using this alternative strategy, break-off at ±25 bp constant loop–anti-sense transitions persisted (Fig 5A, middle panel), leaving the alignable read depth unaffected and not superior to the standard adapter ligation protocol used in subsequent screen replicates (Fig 5A, bottom panel, and S3 Fig).

To align NGS reads to reference shRNA sequences, several software tools are available [11,13,15,21,22]. Preliminary analyses found a high proportion of misalignments in full length read mappings, particularly due to skipped or doubled bases, possibly introduced by the platform technology [22]. To optimize mapping and incorporate incomplete reads, we trimmed total reads to the 22 nucleotides of anti-sense sequence prior to its reference alignment with a zero mismatch tolerance. Aligned read counts were normalized to scale their distributions across screen replicates and sequencing runs, which showed considerable heterogeneity (Fig 5B, left panel). Several normalization methods were evaluated including DESeq, TMM (Trimmed Mean of M-values), Upper Quartile normalization, and Median normalization [23,24]. TMM and DESeq performed similarly and we chose TMM normalization that is implemented in the bioconductor edgeR package [25]. shRNAs with ≥ 50 read alignments in cellular control samples (ctrl_*a*/*b*) were defined as recovered and datasets were filtered accordingly (Fig 5B, right panel).

### Screen performance

Screen performance and data quality are affected by the screening process and constraints of NGS technology and its analysis workflow. The Ion Proton sequencer generated a raw output of 50–75 million reads per run. Using the above alignment strategy with zero mismatch tolerance, between 5.3 and 8.2 million aligned reads were obtained for each screen sample. With a minimum mean coverage of 1,140 aligned reads per each shRNA, the intended read depth of 1,000 reads per shRNA was achieved. Consistent with histograms of ±25 bp read break-off, aligned read counts were lower in first-run test samples and Decode ctrl aliquots compared to screen replicates 1 and 2. Still, the mean percent recovery of shRNAs (defined as shRNAs with ≥ 50 alignments of raw reads) across technical NGS replicates of Decode ctrl was 98% (standard deviation 0.3%), and 94% (standard deviation 3.6%) for screen samples.

The distribution of shRNA abundances affects screen performance, as over- or under-represented shRNAs are more prone to random noise, particularly when screening at lower-fold average shRNA representation. The distribution of shRNA read counts in control samples is expected to reflect the composition of the pooled lentiviral shRNA library, whereas experimental samples are anticipated to gain a broader range of abundances due to altering biological shRNA effects. As shown in box plots of Fig 5B, distributions of raw read counts benefitted from TMM normalization and filtering for shRNAs recovered with ≥ 50 alignments. However, compared to the uniform and close distribution of non-cellular Decode ctrl technical replicates, cellular control samples maintained a broad and heterogeneous read count distribution (Fig 5B, right panel) that provides a more realistic estimate of biological variance including early-onset shRNA effects. Of note, distributions of ctrl_A673 and particularly ctrl_HEK293 were broader in replicate 1 than in their replicate 2 counterparts, pinpointing the limitations of no more than two screen replicates. To relate read count distributions of our dataset to the published dataset of Strezoska *et al*. [12], we calculated the minimum range of shRNA abundance as the minimum fold difference between the least and most abundant shRNAs for 70% of the shRNA population. A less than 10 minimum fold difference in control samples recapitulated published values; with the exception of ctrl_HEK293 replicate 1 showing a broader range (S4 Fig). As expected, the range of abundance of experimental samples was broader with a 14- to 23-fold difference. Here, exp_HEK293 replicate 2 showed a distinct, closer range of distribution (S4 Fig and Fig 5B, right panel). These analyses of shRNA read count distributions via boxplots or the minimum range of abundance provide important insight into data quality and its limitations, such as problematic samples that are to be considered with caution or removed from further analyses.

For evaluation of screen performance in terms of sensitivity, the use of pre-manufactured lentiviral shRNA libraries precludes engineered depletion of defined pool subsets as described in other reports [11,13,15]. Yet, in performing the screen at 100-fold shRNA representation, limitations to screen sensitivity and specificity were deliberate, as discussed above [12]. As a measure of data quality, the reproducibility of shRNA read counts between technical and biological replicates was examined. Pearson correlation coefficients (r) of 0.99 for intra-run and 0.95–0.99 across all inter-run technical replicates of Decode ctrl, in both raw and normalized data, demonstrated excellent technical reproducibility using Ion Proton sequencing. The exp_A673 test sample was also assessed as a technical NGS replicate, with similar inter-run correlation (r = 0.99). Furthermore, its technical replicates undergoing alternative NGS library preparation strategies showed high reproducibility of these libraries (intra- and inter-run r values of 0.98 and 0.97, respectively). Across screen samples, the biological reproducibility of replicates (A673 minimum r = 0.95; HEK293 minimum r = 0.84) and of replicates compared to Decode ctrl (A673 minimum r = 0.70; HEK293 minimum r = 0.67) (S4 Fig) reflected the similarities and heterogeneities of read count distributions described above. Because pooled screening relies on relative changes in shRNA abundance, Pearson correlation values were also calculated for the reproducibility of log2 fold changes of read counts in experimental compared to control samples (Fig 5C). As expected, these coefficients (r values of 0.39 and 0.55) were more strongly affected by replicate heterogeneities, but are in line with values of 0.41 and 0.67 achieved in the highly-optimized screen performed by Strezoska *et al*. [12] at screen depths of 100-fold and 500-fold shRNA representation, respectively.

### Profile filtering for screen hits

#### Online profile filtering tool ProFED

For the determination of screen hits, diverse statistical tests are readily available [4,17,26] but disputable in high-variance settings such as our read count dataset. Therefore, and to facilitate data analysis for the experimental biologist, we opted for a descriptive and interactive approach based on fold changes. Our screen design aims to discover candidate targets that are specific to one of two comparatively screened conditions *a* or *b*, such as distinct oncogene expressions, drug treatments, or other synthetic lethal settings, here modeled through cell lines A673 (condition *a*) and HEK293 (condition *b*). The fold change profile of such screen hits therefore demands a drop in shRNA read count in experimental compared to control sample in one condition, and for this drop to be substantially greater than in the other condition. Defining a hit profile requires an interactive exploration of reasonable fold change thresholds and limits, because these hit thresholds, as in statistical approaches, remain arbitrary. To adapt this for large-scale and heterogeneous datasets and to provide a simple-to-use tool tailored to synthetic lethal shRNA screens, we created ProFED (Profile Filtering of Expression Data), a compact open-source online application that allows the researcher to “slide” through profile thresholds and explore resulting data and filtered hit lists (for websites see Materials and Methods).

**Fig 6:**
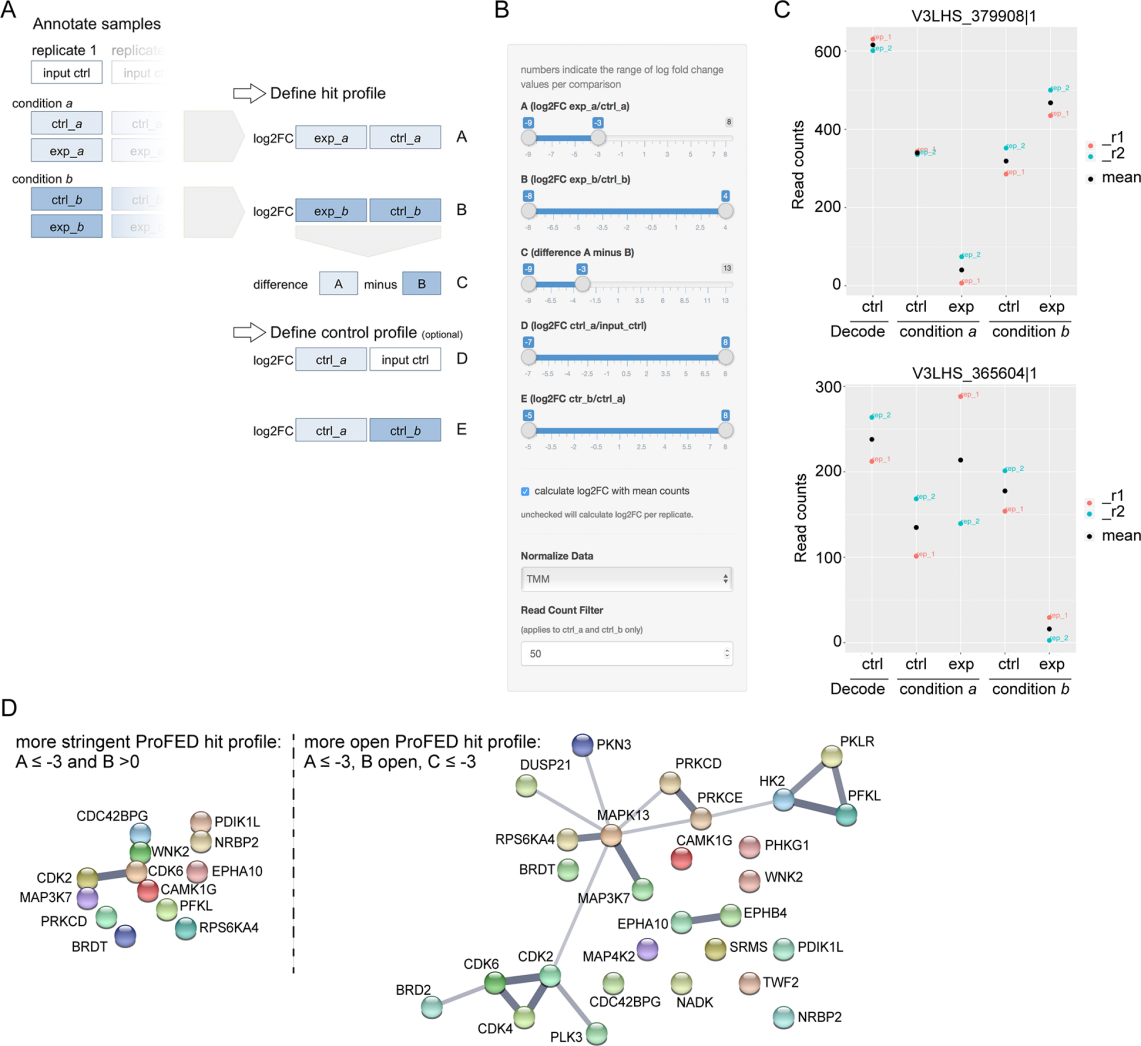
Profile filtering approach for the identification of screen hits. (A) Illustration of sample annotation and basic profile filter parameters. Shading indicates that additional replicates are optional. FC = fold change. (B) Screen-shot from the ProFED online tool showing the “slider” panel used to set fold change thresholds and limits of desired hit profiles. The depicted setting filters for targets specific to condition *a*. (C) Exemplary read count profiles of hits specific to condition *a* (A673; top panel) and *b* (HEK293; bottom panel) as generated by ProFED. (D) STRING analysis of reported and predicted protein-protein interactions among condition *a* (A673)-specific hits. 13 hits filtered with a stringent ProFED profile (Table 1A) were analyzed in comparison to 28 hits based on a more open hit profile (Table 1A plus 1B). Line thickness indicates the strength of data support.

The ProFED profile filtering tool retraces the screen design in comparing two conditions *a* and *b*, each annotated with sets of experimental and control samples (Fig 6A). Multiple screen replicates can be uploaded, normalized, if desired, using TMM or RLE methods, and calculated either individually or as mean. A filter for shRNAs based on a minimum aligned read count in ctrl_*a*/*b* samples is available (Fig 6B). Because sample annotation is left to the researcher, this layout remains open to various modifications and screen constellations. The application translates annotated samples into the profile filtering design and calculates parameters based on log2 fold changes (log2FC) of shRNA read counts (Fig 6A). Parameters A and B describe fold changes in experimental compared to control samples in conditions *a* and *b*, respectively. The difference of this effect in conditions *a* and *b* is calculated as parameter C. In addition to this basic hit profile, an optional control profile (parameters D and E) filters for variance and noise among control samples and a stand-alone input control such as a plasmid or viral library (Fig 6A). After setting these variables, the researcher can slide through fold-change thresholds and their combinations to explore hit profiles and resulting hit lists. Read count plots are generated for each hit to visualize its reproducibility across screen replicates (Fig 6C). Furthermore, to aid quality control and to gain an understanding of count distributions and possible problematic samples, the ProFED tool is equipped with an additional section to explore read count data in box plots, correlation plots, cluster plots, and principal component analyses (see Fig 5B and S4 Fig).

#### Profile filtering of dataset

We utilized the ProFED application to analyze our dataset, which is deposited online as exemplary data. Ctrl_*a*/*b* and exp_*a*/*b* samples of screen replicates 1 and 2 were annotated and data were TMM normalized and filtered for shRNA with ≥ 50 read counts in ctrl_*a*/*b* samples. First, we determined shRNA hits independently for conditions *a* (A673) and *b* (HEK293) by using a log2FC criterion of ≤ -3 in parameters A or B, respectively, corresponding to a ~10-fold drop in shRNA abundance. In condition *a*, 96 and 222 hits were identified in screen replicates 1 and 2, respectively, with an overlap of 25 hits. For condition *b*, these were 273 and 138 hits with an overlap of 30 reproducible hits. This limited overlap indicates a limited reproducibility of hits in our heterogeneous data set. To reduce this “noise” of non-reproducible hits in our hit lists and increase the proportion of reproducible overlap-hits, we opted for an analysis based on mean read counts of both replicates. This approach generated 53 and 61 hits in conditions *a* and *b*, comprising the 25 and 30 reproducible overlap-hits, respectively (S1 Table). Next, we focused on hits specific for either condition *a* or *b*. Several hit profiles were explored using mean read count data. For example, a stringent hit profile demanding a drop in shRNA abundance by a log2FC of ≤ -3 in one condition versus no drop (log2FC > 0) in the other revealed 13 hits specific to condition *a* (Table 1A), and 10 hits specific to condition *b*. A more open setting that released the second parameter but applied parameter C to maintain a difference between conditions *a* and *b* of at least 3 log2FC (exemplified in Fig 6B), generated an additional 15 and 17 hits specific to conditions *a* (Table 1B) and *b*, respectively. Fig 6C shows exemplary read count plots of such condition-specific hits, and ProFED hit lists for all exemplary hit profiles are provided in S1 Table. In several more profile settings combining parameters A-C and control profiles, both the ProFED tool and manual analyses produced identical hit lists. Using ProFED, the instant generation of read count plots proved a helpful feature in the evaluation of hit quality and reproducibility, particularly following the identification of problematic samples as described above. A limited reproducibility of hits is not unexpected here, given the chosen technical screen parameters (100-fold average shRNA representation in no more than two biological replicates) and their performance discussed above. However, it underlines that when aiming to establish a complete target profile, screening strategies using higher (≥ 500-fold) representation remain more suitable. Nevertheless, our primary aim to discover (any promising) condition-specific target hits was achieved and our data therefore encourage resource-saving approaches with 100-fold representation (in at least three replicates) for such purposes.

**Table 1:** Screen hits specific to the A673 cell line (condition *a*) as filtered using ProFED.

	Gene symbol	Gene name	ProFED parameter			Reference ^a^
			A	B	C	
**A**	**Hit list of more stringent ProFED profile: A ≤ -3 and B > 0**					
	EPHA10	EPH receptor 10	-6.04	0.01	(-6.06)	
	WNK2	WNK lysine deficient protein kinase 2	-3.66	2.11	(-5.77)	
	CAMK1G	calcium/calmodulin dependent protein kinase IG	-4.42	1.15	(-5.56)	
	BRDT	bromodomain testis associated	-3.34	1.70	(-5.04)	
	PFKL	phosphofructokinase, liver type	-3.24	1.57	(-4.81)	
	CDK6	cyclin dependent kinase 6	-3.01	1.77	(-4.78)	[27]
	RPS6KA4	ribosomal protein S6 kinase, polypeptide 4	-4.24	0.04	(-4.64)	
	CDC42BPG	CDC42 binding protein kinase gamma	-3.17	0.99	(-4.16)	
	PDIK1L	PDLIM1 interacting kinase 1 like	-3.09	0.83	(-3.92)	[28]
	MAP3K7	mitogen-activated protein kinase kinase kinase 7	-3.08	0.76	(-3.84)	
	CDK2	cyclin dependent kinase 2	-3.66	0.09	(-3.75)	[28,29]
	NRBP2	nuclear receptor binding protein 2	-3.11	0.55	(-3.65)	
	PRKCD	protein kinase C delta	-3.19	0.26	(-3.45)	
**B**	**Additional** **hits with more open ProFED profile: A ≤ -3, B open, C ≤ -3**					
	DUSP21	dual specificity phosphatase 21	-6.20	(-0.27)	-5.93	[30]
	EPHB4	EPH receptor B4	-5.74	(-0.43)	-5.31	
	SRMS	SRC-related kinase lacking C-terminal regulatory kinase and N-terminal myristilation sites	-5.80	(-0.63)	-5.17	
	MAP4K2	mitogen-activated protein kinase kinase kinase kinase 2	-5.81	(-0.76)	-5.05	
	MAPK13	mitogen-activated protein kinase 13	-5.69	(-0.71)	-4.98	[28]
	PRKCE	protein kinase C epsilon	-4.73	(-0.20)	-4.53	
	PKLR	pyruvate kinase L/R	-4.60	(-0.36)	-4.24	[28]
	HK2	hexokinase 2	-4.06	(-0.40)	-3.65	
	BRD2	bromodomain containing 2	-3.92	(-0.43)	-3.49	
	CDK4	cyclin dependent kinase 4	-5.51	(-2.02)	-3.48	[28,31]
	PLK3	polo like kinase 3	-3.97	(-0.51)	-3.46	
	PKN3	protein kinase N3	-4.17	(-0.78)	-3.40	
	PHKG1	phosphorylase kinase catalytic subunit gamma 1	-3.96	(-0.70)	-3.26	
	NADK	NAD kinase	-3.79	(-0.59)	-3.20	
	TWF2	twinfilin actin binding protein 2	-3.07	(-0.03)	-3.04	

^a^ previously reported as therapeutic target or respective screen hit in Ewing sarcoma.

Exploring hit profiles with ProFED, our exemplary hit lists demonstrate that the flexible profile filtering approach can determine both strong hits and their broader hit spectrum (exemplified in Table 1A and 1B, respectively), which in turn can underscore these hits through multiple shRNAs hits per gene or facilitate hit classification using cellular network and gene ontology platforms. As illustrated in Table 2 and Fig 6D for a stringent compared to a more open hit profile (13 versus 28 hits as defined in Table 1), a broader hit spectrum with an increased number of hits facilitated identification of enriched gene ontologies and protein-protein interactions among target hits specific to the A673 Ewing sarcoma cell line (condition *a*); which revealed, in addition to kinase-inherent phosphorylation processes, an enrichment of kinases involved in cell cycle progression and glycolysis. Still, it remains for all candidate hits of shRNA screens that validation of their involvement in cell viability or respective phenotypes screened is essential because efficacies of gene silencing and off-target effects continue to pose central limitations to RNA interference-based approaches [8,16]. While this topic is beyond the scope of this assay development study, some of the hits identified here have previously been reported as potential therapeutic targets or respective screen hits, as displayed in Table 1 for A673 (condition *a*)-specific hits.

**Table 2:** Enriched gene ontologies of A673 (condition *a*)-specific hits.

	Term	# of genes	Enrichment	p-value
**A**	**Hit list of more stringent ProFED profile: A ≤ -3 and B > 0** ^a^			
	GO:0006468 ~protein phosphorylation	8	22.7	7.30E-09
	GO:0035556 ~intracellular signal transduction	5	16	1.40E-04
	GO:0016572 ~histone phosphorylation	3	387.5	2.10E-05
**B**	**Hit list of more open ProFED hit profile: A ≤ -3, B open, C ≤ -3** ^b^			
	GO:0006468 ~protein phosphorylation	14	18.4	5.20E-14
	GO:0035556 ~intracellular signal transduction	9	13.4	1.50E-07
	GO:0018105 ~peptidyl-serine phosphorylation	5	24	4.50E-05
	GO:0000082 ~G1/S transition of mitotic cell cycle	4	23.5	5.70E-04
	GO:0051301 ~cell division	4	6.9	1.80E-02
	GO:0016572 ~histone phosphorylation	3	179.9	1.10E-04
	GO:0061621 ~canonical glycolysis	3	69.2	7.90E-04
	GO:0018108 ~peptidyl-tyrosine phosphorylation	3	11.8	2.50E-02
	GO:0007049 ~cell cycle	3	8.3	4.70E-02

Biological process; p <0.05 and > 3 genes per category.

^a^ corresponding to Table 1A (13 hits).

^b^ corresponding to Table 1A plus 1B (28 hits).

### Conclusions

Here we describe a target discovery screen using pooled shRNA libraries and Ion Proton NGS-based deconvolution, in a comparative screen design that is suitable to identify tumor cell-specific targets and synthetic lethal dependencies. We demonstrate that these screens can be successful from the first assay performance and without specialized screening units, although assay development of this multi-step procedure remains challenging. We present a complete workflow model that can aid in this process, encompassing each step from cell-based assay to analytical algorithm and their experimental and theoretical aspects. As part of our analytical algorithm, we created an open-source tool that facilitates the descriptive analysis of heterogeneous datasets, such as those generated from lower-depth pooled screens, for which automated and non-interactive statistical methods remain debatable. Its profile filtering approach to hit calling and a versatile design provide a fast and easy pipeline for the analysis of shRNA and other count-based datasets that can complement unsupervised statistical methods. Of note, many of the methodological challenges and solutions presented here for pooled shRNA screening are shared with the CRISPR/Cas precise genome-editing technology [9,32], which is emerging as an alternative approach to functional genomics screening.

## Materials and methods

### Cell culture

The Ewing sarcoma cell line A673 was a kind gift of Dr. S. Lessnick (Nationwide Children’s Hospital, Columbus, OH; Feb 2014) and contained (for prospective synthetic lethal screens) a retroviral pSRP shRNA expression vector with control insert [33]. The human embryonic kidney cell line HEK293 (ACC 305; DSMZ, Braunschweig, Germany) was from our institutional repository. Both cell lines were authenticated by short tandem repeat examination after pooled screens to confirm cell line identity in screen hits. The HEK293T packaging cell line was purchased from Thermo Fisher Scientific (Cat-No. HCL4517; Waltham, MA; Sept 2012). All cell lines were cultured at 37°C with 5% CO_2_ in DMEM high-glucose medium supplemented with 10% fetal bovine serum (FBS) and for HEK293T with 20 mM HEPES (AppliChem, Darmstadt, Germany). All cell lines were regularly tested to be free of mycoplasma.

### shRNA plasmids

GIPZ^TM^ lentiviral shRNA plasmids and packaging plasmids (Trans-Lentiviral Packaging Kit, Cat-No. TLP5912) were purchased from GE Healthcare Dharmacon (Lafayette, CO). Plasmids containing non-silencing shRNA (Cat-No. RHS4346) and control shRNAs targeting GAPDH (Cat-No. RHS4371) and LaminA/C (clone V2LHS_62719) were obtained as glycerol stocks and prepared according to the manufacturer’s protocol. Inserts were sequence-confirmed prior to use. Non-silencing (Cat-No. RHS4348) and LaminA/C (V2LHS_62719) shRNAs were also purchased as lentiviral particles. For detailed information on the GIPZ system see http://dharmacon.gelifesciences.com/uploadedFiles/Resources/decode-pooled-lentiviral-shrna-screening-libraries-manual.pdf.

### Lentiviral shRNA packaging

For generation of GIPZ shRNA lentivirus, 5 x10^6^ HEK293T packaging cells were seeded into 10 cm tissue culture dishes. On the following day, 40 μg of GIPZ shRNA plasmid and 10 μg of packaging plasmids were diluted to 450 μl in sterile water. 50 μl of 2.5 M CaCl_2_ solution were added, followed by 500 μl 2x HBSS under constant air bubbling with a pipette. This transfection solution was incubated for 15 min. Growth medium was replaced by 5 ml DMEM containing 10% FBS, 20 mM HEPES and 25 mM chloroquine and transfection solution was added drop-wise. Cells were incubated for 6 h before transfection medium was replaced by 5 ml of standard growth medium. Lentiviral supernatant was harvested after 24 h, stored at 4°C, and again after 48 h. Supernatants were combined and filtered through a 0.45 μm syringe filter (Corning, Corning, NY). Virus was concentrated using 2.5 ml of 5x PEG*-it* solution (System Biosciences, Mountain View, CA) according to the manufacturer’s protocol. Virus concentrate was resuspended in 1 ml DMEM and stored at 4°C for up to 1 week.

### Lentiviral shRNA transduction

Spin transduction were performed in 6-well plates, due to the centrifuge capacities of our Biosafety Level 2 containment facility. Target cells were seeded one day previously at a density of 10.5 (A673) and 7.5 (HEK293) x10^3^ cells per cm^2^. For transduction, growth medium was replaced with concentrated shRNA lentivirus or purchased particles diluted to 2 ml volume in DMEM containing 10% FBS and 8 μg / ml polybrene (Merck, Darmstadt, Germany). 6-well plates were then centrifuged at 1,000 g at 37°C for 1 h before incubation at 37°C with 5% CO_2_. After 24 h, virus was replaced with standard growth medium.

### Virus titration

Cells were seeded into 24-well plates at above densities. One day later, one well was counted to determine cell number. Other wells were transduced with serial dilutions of lentivirus as outlined above in a total volume of 200 μl. After 24 h, 800 μl of standard growth medium was added. Transduced cell fractions were determined after 72 h. The functional titer expressed in transducing units per ml [TU / ml] was calculated as: (GFP-positive cell fraction x total cell number at transduction x dilution factor) ÷ transduction volume. Calculations were performed for four dilution factors of the serial dilution and three independent experiments to determine mean functional titers. Functional titers of purchased non-silencing shRNA lentivirus served to calculate the relative transduction efficiency of A673 and HEK293 target cell lines as: functional titer [TU / ml] ÷ virus-batch reference titer [TU / ml]. The anticipated titer of the Decode Pooled Lentiviral shRNA Library was deduced as: relative transduction efficiency x reference titer [TU / ml].

### Flow cytometry

GIPZ plasmids contain the TurboGreen-Fluorescent-Protein (GFP) reporter as part of a bicistronic transcript with shRNA inserts. For flow cytometry analysis of GFP expression, cells were washed in PBS and fixed in 4% paraformaldehyde. GFP fluorescence was measured using a FACS Canto II cytometer (BD Bioscience, Franklin Lakes, NJ) and its FACS Diva software. Flow cytometry plots were generated using FlowJo v10 software (FlowJo LLC, Ashland, Oregon).

### Western blotting

Cell lysis, procedures, and buffers were as previously described [34]. LaminA/C antibody (aa 398–490; mouse monoclonal; 1:1000 dilution) was from BD Transduction Laboratories (Cat-No. 612162; Heidelberg, Germany) and actin (C4; goat polyclonal; 1:1000 dilution) was from Santa Cruz Biotechnology (Cat-No. sc-47778; Santa Cruz, CA).

### Pooled screen and cell sampling

The Decode^TM^ Pooled Lentiviral Protein Kinase shRNA Library (Cat-No. RHS6078; GE Healthcare Dharmacon) consisting of one pool of 4,675 shRNAs was purchased as ready-to-use lentiviral particles. Transductions were performed such that on average 100 copies of each shRNA were integrated into target cells. This was performed at a MOI of 0.5 that predicts shRNA integration into 39% of exposed cells. These parameters required 1.2 x10^6^ cells based on 4,675 shRNAs x 100 integration events ÷ 0.39 cells with shRNA integration. In turn, this needed 0.6 x10^6^ lentiviral transducing units (TU), given 1.2 x10^6^ cells x 0.5 MOI. Because anticipated titers of the pooled shRNA library were not significantly different for A673 and HEK293, a titer of 120 x10^6^ TU / ml was applied for both cell lines. Hence, 0.6 x10^6^ TU ÷ 120 x10^6^ TU / ml = 5 μl of viral particles were used per 1.2 x10^6^ exposed cells.

Spin transduction was performed in a 6-well format as described above. Cells of one well were counted and the required number of wells and amount of virus particles per well were scaled accordingly. To account for variance in transduction efficiency and toxicity, additional wells were incorporated as a backup. Puromycin (Sigma Aldrich, Munich, Germany) selection with 1 μg / ml was initiated 72 h after transduction and continued throughout the experiment. After 48 h of selection, cell populations were divided into early time-point (day 5) control samples (ctrl_*a*/*b*) and continued cultures. At this time-point and throughout the screen, aliquots of all cultured and collected populations were assessed by flow cytometry to confirm at least 467,500 GFP-positive cells corresponding to a 100-fold shRNA representation at single shRNA copy integration per cell. For ctrl_*a*/*b*, absolute numbers of collected GFP-positive cells were documented and pellets were frozen at -80°C. Cultured cell populations were transferred to T75 flasks and passaged at 70–80% confluence to maintain logarithmic growth and avoid localized growth restrictions. After another 5 passages (day 23), cells of experimental samples (exp_ *a*/*b*) were frozen as described above. The screen was performed in three independent biological replicates. To minimize variability, we used same-passage cells and consistent batches of media, supplements, and tissue culture plates. In all subsequent steps, samples of each replicate were handled in parallel, while independent replicates were processed consecutively.

### Genomic DNA isolation and shRNA PCR amplification

The amount of gDNA required to maintain the average shRNA fold representation throughout PCR amplification can be calculated based on the corresponding cell number and mass of a human genome. However, this is inaccurate in cell lines with complex karyotypes such as A673 [35] and many other cancer cell lines. Therefore, gDNA was isolated from frozen cell pellets (QiAMP DNA Mini Kit; Qiagen, Hilden, Germany) and assessed for amount and purity using spectrophotometry (NanoDrop 2000 Spectrophotometer; Thermo Fisher Scientific). Based on documented numbers of GFP-positive cells per sample, gDNA amounts equivalent to 467,500 GFP-positive cells were used to PCR amplify the anti-sense shRNA region. Multiple parallel reactions were performed. Each 50 μl reaction contained 800 ng template gDNA, 200 μM dNTP, 0.5 μM primers, 1 M betaine, 1.5 μl DMSO and 2 units Phusion HotStart II Polymerase in 1x Phusion HF buffer. Primers were 5’-TAGTGAAGCCACAGATGTA (forward) and 5’-GTATCAAAGAGATAGCAAGG (reverse). PCR was performed at 98°C for 1.5 min followed by 33 cycles of 98°C (10 s), 56°C (30 s), 72°C (30 s), and final 72°C (10min) on a TProfessional cycler (Biometra, Göttingen, Germany). Parallel reaction products were pooled and purified using the GeneJET PCR Purification Kit (Thermo Fisher Scientific). Product quantity and quality were evaluated using NanoDrop spectrophotometry, Qubit fluorometric quantification (Qubit 3.0 and dsDNA high-sensitivity assay; Thermo Fisher Scientific) and Bioanalyzer gel electrophoresis (Bioanalyzer 2100 and Agilent DNA 1000 Kit; Agilent Technologies, Santa Clara, CA).

For the Decode ctrl viral library control, lentiviral RNA from 467,500 transducing units was isolated using the GeneJET viral DNA and RNA purification Kit (Thermo Fisher Scientific) according to the manufacturer’s protocol. Transcription to cDNA was performed with random hexamers and M-MLV reverse transcriptase (Promega, Madison, WA). Total cDNA was PCR amplified in multiple parallel reactions containing 600 ng of template per 50 μl. The PCR protocol and product purification and evaluation were as described above.

For confirmatory sequencing of non-silencing shRNA PCR product, 2 μl of template was amplified in a 10 μl reaction containing 1.4 μl buffer, 0.6 μl Reaction Mix (BigDye Terminator v3.1; Thermo Fisher Scientific) and 1 μM primer 5’-GTATCAAAGAGATAGCAAGG. Sequencing PCR was performed on a TProfessional cycler with initial denaturation (96°C, 4 min) and 35 cycles of 96°C (20 s), 50°C (10 s), and 60°C (2 min). Following Sephadex purification (GE Healthcare), capillary-electrophoresis was performed on an Applied Biosystems 3730xl DNA Analyzer (Thermo Fisher Scientific).

### Ion Proton library preparation and NGS

Ion Proton NGS libraries were prepared from 500 ng of purified shRNA anti-sense PCR product using the KAPA Library Preparation Kit for Ion Torrent Platforms (Cat-No. KK8301; KAPA Biosystems, Wilmington, MA) according to the manufacturer’s protocol. Adapter P1 and barcoded A adapter concentrations (Cat-No. KK 8331) were as suggested for 130 bp median fragment size. Post-ligation cleanup was performed with double-sided size selection for 200 bp target size fragments and library amplification was with 8 cycles. Final products were quantified using Qubit 3.0 fluorometric assay (dsDNA HS assay) and confirmed for fragment size using Bioanalyzer electrophoresis (Agilent High Sensitivity DNA Kit). Barcoded samples were diluted to 100 pM concentrations and one screen replicate plus Decode ctrl aliquot were multiplexed per one NGS run. Sequencing was performed on an Ion Proton platform using the Ion PI^TM^ Chip Kit v3 with Ion PI^TM^ Hi-Q^TM^ OT2 200 and Ion PI^TM^ Hi-Q^TM^ Sequencing 200 Kits.

### Bioinformatics analysis

The Ion Torrent software performed de-multiplexing of sample barcodes. Sequence read lengths ranged from 8 to 385 bp. Based on constant loop and 3’ vector nucleotide sequences, we made a strategic trimming of reads to the 22 nucleotides of anti-sense sequence using cutadapt software [36] and fastx toolkit (http://hannonlab.cshl.edu/fastx_toolkit/). Read alignment was performed using bowtie [21] with a zero mismatch stringency. Python and R scripts were applied to extract read counts from aligned sequence files. Normalization of read counts was performed using the TMM (Trimmed Mean of M-values) normalization method that is implemented in the bioconductor edgeR package [25]. The minimum range of abundance was calculated based on those 70% of shRNAs that minimized the fold difference between the most and least abundant shRNA. Box plots and Pearson correlations were computed using R base package. All three calculations were performed on raw read counts and on TMM normalized read count data filtered for shRNAs that were defined as recovered, based on ≥ 50 read alignments in ctrl_*a*/*b* samples.

Gene ontology analyses were performed using the database for annotation, visualization and integrated discovery (DAVID) v6.8 [37]. Analyses of reported and predicted protein-protein interaction were performed using the STRING platform v10.5 and based on text mining, experiments and databases, with a minimum interaction score of medium confidence (0.40) [38].

### ProFED profile filtering tool

Data analysis was performed descriptively based on log2 fold changes of normalized read counts as outlined in Results and Discussion. To filter shRNAs for hits according to fold change profiles, we created the ProFED open-source online tool using RShiny, a web application for R (http://shiny.rstudio.com/). For mathematical formulations see S1 Appendix. The ProFED analysis package processes diverse expression data uploaded in csv-format. Our screen data was deposited as an exemplary dataset online. The application is available online at http://ebi056.uni-muenster.de:3838/profed/ and can be downloaded from GitHub (https://github.com/korpleul/PONED) or our institutional website (http://complex-systems.uni-muenster.de/sinfo.html). http://complex-systems.uni-muenster.de/sinfo.html

## Supporting information

S1 Fig Additional figure on target cell transduction and selection. (A) Representative flow cytometry scatter plots of A673 and HEK293 cells demonstrating GFP-positive and -negative cell populations 96 h after transduction with non-silencing shRNA. (B) Puromycin dose-response curve of native non-transduced A673 (left) and HEK293 (right) cells. Viable cells were counted by trypan-blue exclusion method. Bar graphs represent mean ±SD of three independent experiments.
(TIF)

S2 Fig Additional Bioanalyzer electrophoresis profiles. Bioanalyzer electrophoresis profiles of (A) purified PCR products generated from Decode ctrl and a representative test sample (corresponding to Fig 4D), and of (B) purified PCR products and (C) Ion Proton NGS libraries generated from screen replicate 1 (corresponding to Fig 4E). FU = fluorescent units; asterisk (*) indicates markers; arrow indicates expected fragment size.
(TIF)

S3 Fig Additional Ion Proton read length histograms. (A) Read length histogram of a technical NGS replicate of exp_A673 (corresponding to Fig 5A, top panel). (B) Bioanalyzer electrophoresis profile of an Ion proton NGS library (exp_A673 test sample) generated in an alternative strategy incorporating barcodes and platform adapters in an additional 16-cycle PCR (corresponding to Figs 4D and 5A, middle panel). (C) Read length histograms of screen replicate 1, where mean and median read lengths approached the target read length of 127 bp (corresponding to Fig 5A, bottom panel).
(TIF)

S4 Fig Additional figure on shRNA read count distribution and reproducibility. (A) The minimum range of shRNA abundance, calculated as the minimum fold difference between the least and most abundant shRNAs for 70% of the shRNA population [12]. r1 and r2 indicate screen replicates 1 and 2, respectively. (B) Scatter plot matrix and Pearson correlation coefficients for screen replicates 1 and 2. Both calculations (A and B) were performed on TMM normalized data sets filtered for shRNAs with ≥ 50 read counts in ctrl_*a*/*b* samples.
(TIF)

S1 Table Hit lists generated using the ProFED online application. These hit lists refer to the exemplary hit profile criteria described in Results and Discussion.
(XLSX)

S1 Appendix ProFED Workflow. Mathematical formulations underlying the ProFED tool.
(PDF)
