# Supplementary figures and images for: Target discovery screens using pooled shRNA libraries and next-generation sequencing: A model workflow and analytical algorithm
Source: PLoS One. 2018 Jan 31;13(1):e0191570. doi: 10.1371/journal.pone.0191570 (PMC5792015; doi:10.1371/journal.pone.0191570)

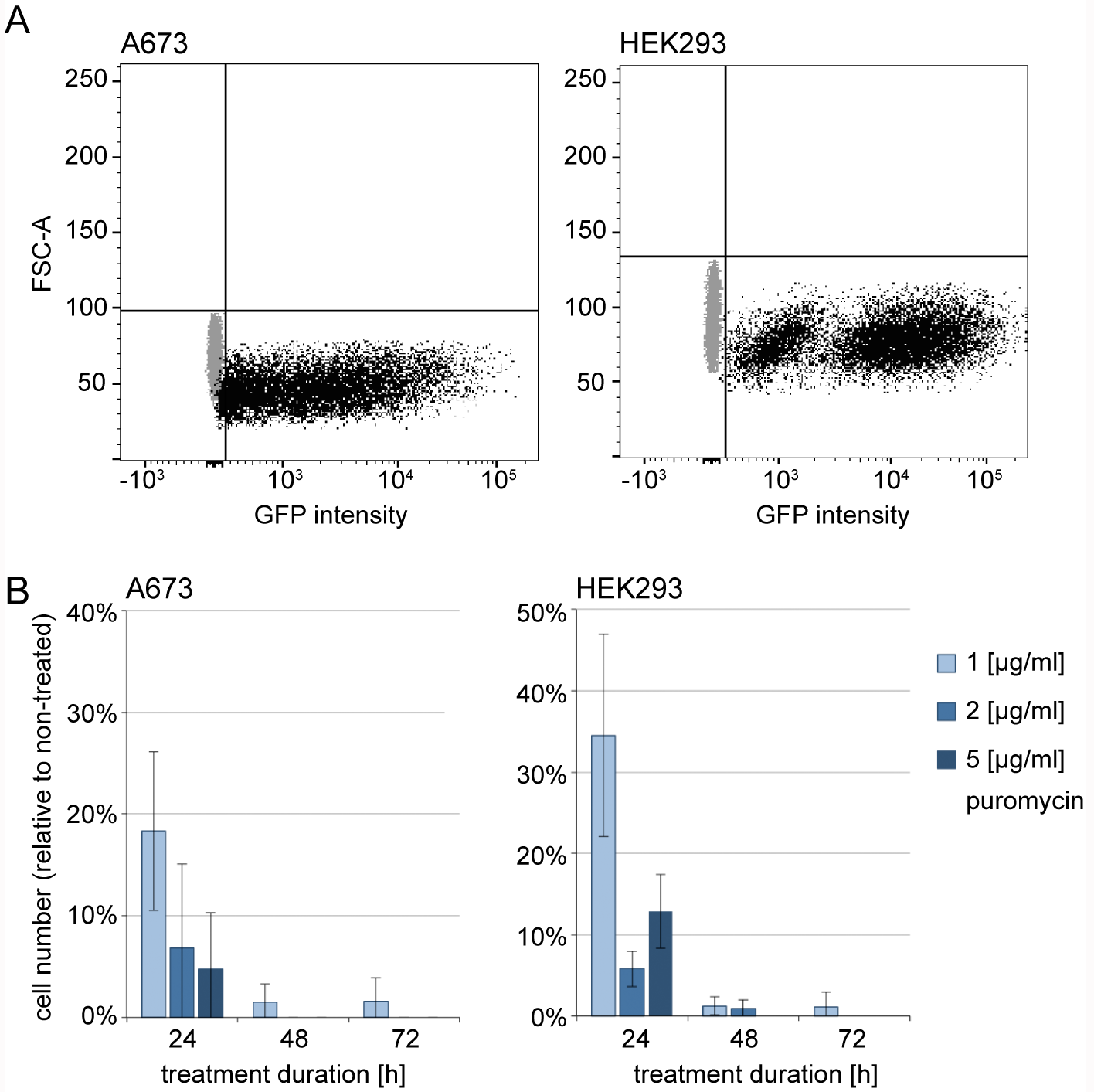

Supplement: S1 Fig — (A) Representative flow cytometry scatter plots of A673 and HEK293 cells demonstrating GFP-positive and -negative cell populations 96 h after transduction with non-silencing shRNA. (B) Puromycin dose-response curve of native non-transduced A673 (left) and HEK293 (right) cells. Viable cells were counted by trypan-blue exclusion method. Bar graphs represent mean ±SD of three independent experiments. (TIF) [file pone.0191570.s001.tif]

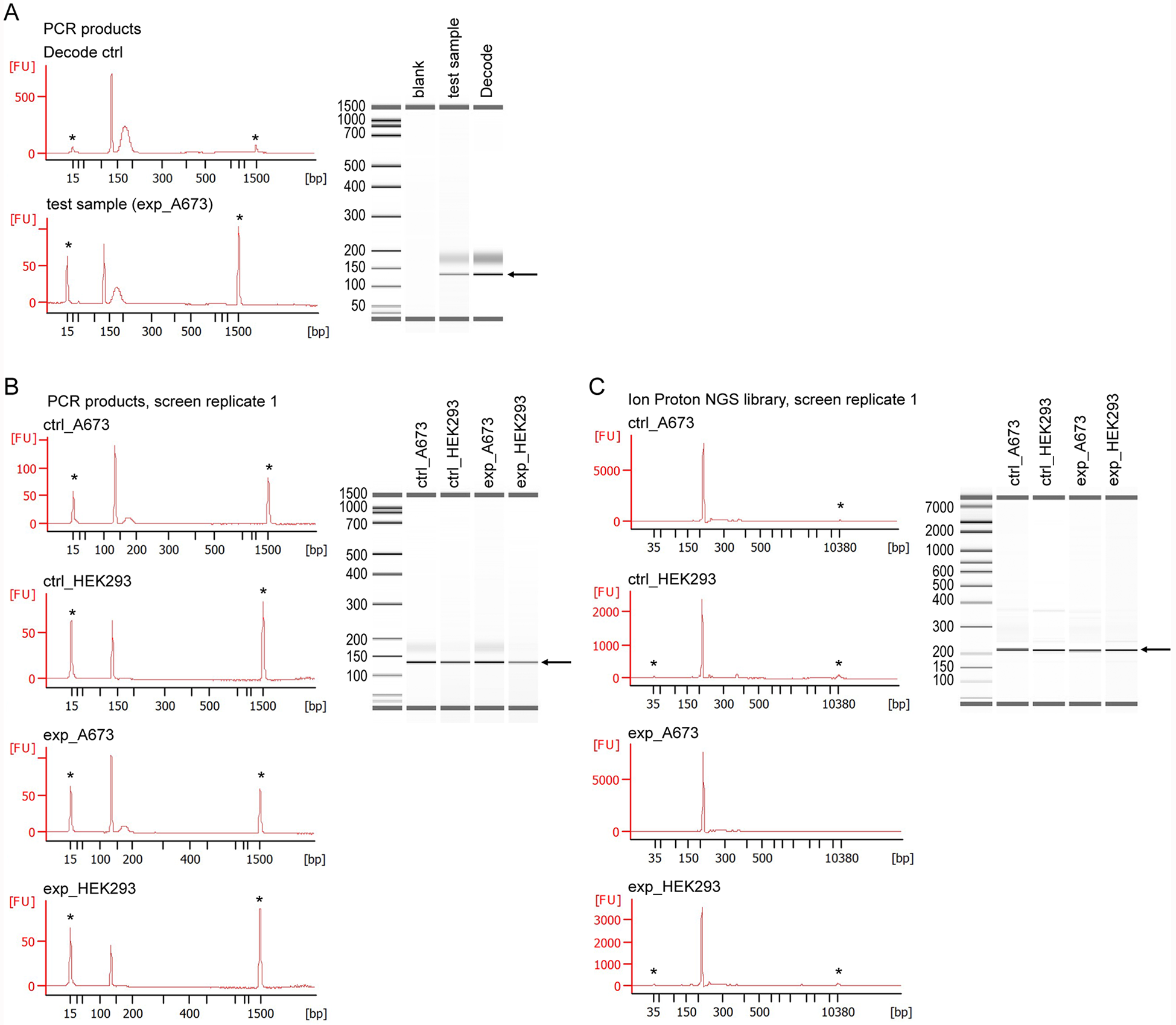

Supplement: S2 Fig — Bioanalyzer electrophoresis profiles of (A) purified PCR products generated from Decode ctrl and a representative test sample (corresponding to Fig 4D), and of (B) purified PCR products and (C) Ion Proton NGS libraries generated from screen replicate 1 (corresponding to Fig 4E). FU = fluorescent units; asterisk (*) indicates markers; arrow indicates expected fragment size. (TIF) [file pone.0191570.s002.tif]

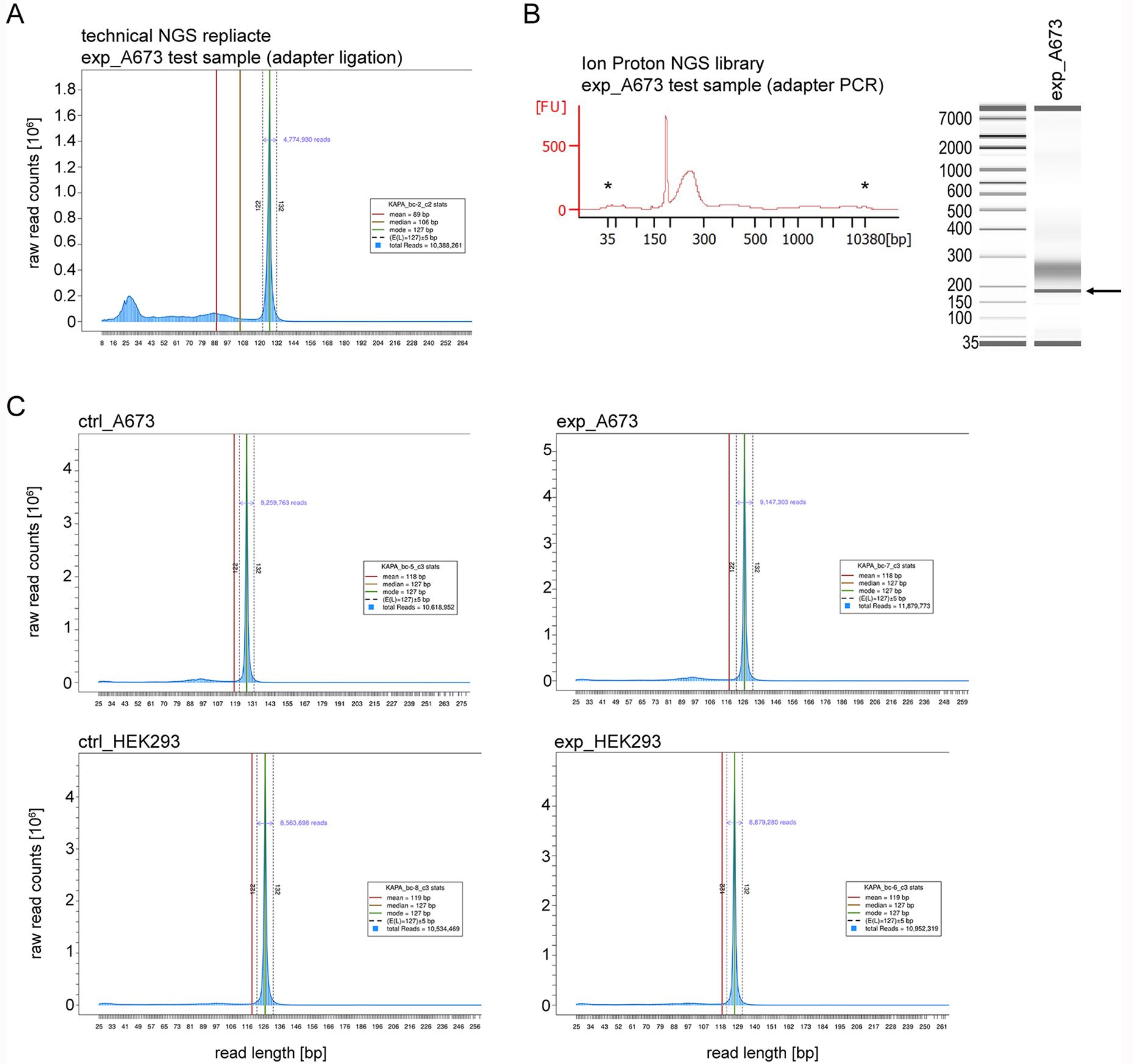

Supplement: S3 Fig — (A) Read length histogram of a technical NGS replicate of exp_A673 (corresponding to Fig 5A, top panel). (B) Bioanalyzer electrophoresis profile of an Ion proton NGS library (exp_A673 test sample) generated in an alternative strategy incorporating barcodes and platform adapters in an additional 16-cycle PCR (corresponding to Figs 4D and 5A, middle panel). (C) Read length histograms of screen replicate 1, where mean and median read lengths approached the target read length of 127 bp (corresponding to Fig 5A, bottom panel). (TIF) [file pone.0191570.s003.tif]

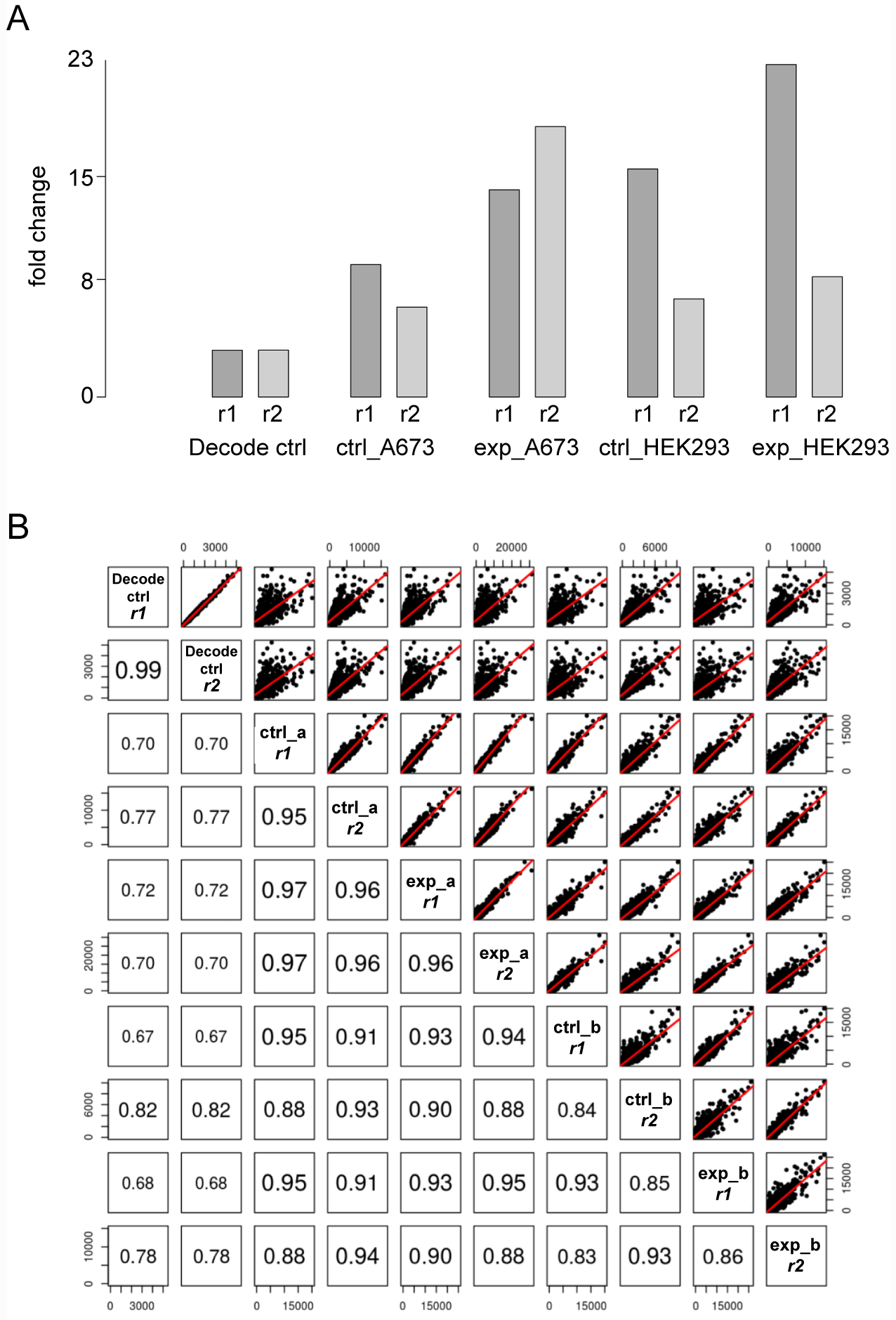

Supplement: S4 Fig — (A) The minimum range of shRNA abundance, calculated as the minimum fold difference between the least and most abundant shRNAs for 70% of the shRNA population [12]. r1 and r2 indicate screen replicates 1 and 2, respectively. (B) Scatter plot matrix and Pearson correlation coefficients for screen replicates 1 and 2. Both calculations (A and B) were performed on TMM normalized data sets filtered for shRNAs with ≥ 50 read counts in ctrl_a/b samples. (TIF) [file pone.0191570.s004.tif]
