# Supplementary material for: Target discovery screens using pooled shRNA libraries and next-generation sequencing: A model workflow and analytical algorithm
Source: PLoS One. 2018 Jan 31;13(1):e0191570. doi: 10.1371/journal.pone.0191570 (PMC5792015; doi:10.1371/journal.pone.0191570)
Supplement: S1 Appendix — Mathematical formulations underlying the ProFED tool. (PDF) [file pone.0191570.s006.pdf]

S1 Appendix. ProFED Workflow

Introduction

This document is to detail the workflow underlining the ProFED web application filtering functionality. The analysis panel of ProFED contains input fields for entering the samples. The input fields are labelled `input_ctrl`, `ctrl_a`, `exp_a`, `ctrl_b`, `exp_b`. They take in selected columns of the data matrix as an input. Where `input_ctrl` are annotated with optional decode library controls; `ctrl_a` and `ctrl_b` are annotated with the control samples; `exp_a` and `exp_b` are annotated with the experimental samples per respective cell lines.

1 – Calculation of the parameters

The data that is fed into the ProFED application is a data matrix. The rows of the matrix indicate the features or genes or row IDs. The columns of the matrix indicate the sample names. The data matrix is expected to be populated with natural numbers which denote to the read counts per respective sample per respective row ID. Assuming that all the input fields are populated with appropriately selected columns of the data matrix, where more than one selected column per field would be considered as an experimental replicate designated to that input field. Assuming that each of the input fields have equal number of replicates `R` and each of the replicate has equal number of elements (features or genes) `W` , the parameters `A`, `B`, `C`, `D`, `E` are calculated by the function $f\left((m_{ij}),(n_{ij})\right)$.

Define

$$exp\_a, ctrl\_a, exp\_b, ctrl\_b, input\_ctrl \in \mathbb{R}^{W\times R} \;, \tag{1}$$

$$exp\_a := (ea_{ij}) \;, \tag{1a}$$
$$ctrl\_a := (ca_{ij}) \;, \tag{1b}$$
$$exp\_b := (eb_{ij}) \;, \tag{1c}$$
$$ctrl\_b := (cb_{ij}) \;, \tag{1d}$$
$$input\_ctrl := (ic_{ij}) \;. \tag{1e}$$

Now, define

$$f : \mathbb{R}^{W\times R} \times \mathbb{R}^{W\times R} \to \mathbb{R}^{W\times R} \tag{2}$$

by

$$f\left((m_{ij}),(n_{ij})\right) = \left(log_2\left(\frac{m_{ij}}{n_{ij}}\right)\right) \;. \tag{3}$$

Let

$$f((ea_{ij}),(ca_{ij})) = A \tag{4}$$
$$f((eb_{ij}),(cb_{ij})) = B \tag{5}$$
$$A - B =: C \tag{6}$$
$$f((ca_{ij}),(ic_{ij})) = D \tag{7}$$
$$f((cb_{ij}),(ca_{ij})) = E \tag{8}$$

Calculation with the mean of replicates

In the case where, 1) the user has chosen the option *calculate log2FC with mean counts* or 2) the user enters unequal number of replicates for each of the input fields, calculation of the parameters will be done with the mean of the data columns selected for the corresponding input fields.

Let *Z* be a matrix made of an entry from an input field containing *R* number of replicates, where *R* are the number of columns and *W* are the number of rows. The calculation of mean is defined by the function $\lambda(Z)$.

For

$$(z_{ij}) =: Z \in \mathbb{R}^{W \times R} \tag{9}$$

define

$$\lambda : \mathbb{R}^{W \times R} \to \mathbb{R}^{W \times 1} \tag{10}$$

by

$$\lambda(z)_i = \left(\frac{\sum_{j=1}^{R} z_{ij}}{R} \right) . \tag{11}$$

2 – Filtering for hits

The final result table is filtered based on the user-defined limits for the calculated parameters from *A* to *E*. In the ProFED application, we provide two result tables based on the usage of the optional *input_ctrl* field in the analysis panel.

Let $L_1, L_2 \in \mathbb{R}$ be the user-defined minimum and maximum threshold values for filtering the respective parameters *A* to *E*. The set of the row indices that fall into the defined limits is calculated as a function that takes a parameter matrix $X^{W \times R}$and the limits L_1, L_2 as the arguments.

Define

$$\beta_{L_1,L_2} : X^{W\times R} \to \wp(\mathbb{N}) \text{ , where } \wp \text{ is a power set} \tag{12}$$

$$\beta_{L_1,L_2}((x_{ij})) = \{i : \gamma_{L_1,L_2}(x_{i\cdot}) == R\} \tag{13}$$

where

$$\gamma_{L_1,L_2} : X^R \to \mathbb{R} \tag{14}$$

$$\gamma_{L_1,L_2}(x) = \sum_{j=1}^{R} \delta_{L_1,L_2}(x_j) \tag{15}$$

and

$$\delta_{L_1,L_2} : \mathbb{R} \to \{0,1\} \tag{16}$$

$$\delta_{L_1,L_2}(a) = \begin{cases} 1, & \text{if } L_1 \le a \le L_2 \\ 0, & \text{otherwise.} \end{cases} \tag{17}$$

The hit indices H are then defined by the intersection of the filtered indices for each of the calculated parameters. I_D will be an independent hit indices as it relies on the optional *input_ctrl* input field.

$$H = I_A \cap I_B \cap I_C \cap I_E \tag{18}$$

where

$$\beta_{L_{a1},L_{a2}}(A) =: I_A \tag{18a}$$
$$\beta_{L_{b1},L_{b2}}(B) =: I_B \tag{18b}$$
$$\beta_{L_{c1},L_{c2}}(C) =: I_C \tag{18c}$$
$$\beta_{L_{d1},L_{d2}}(D) =: I_D \tag{18d}$$
$$\beta_{L_{e1},L_{e2}}(E) =: I_E \tag{18e}$$
